# Response to Comments on Meo *et al*. Association of Exposure to Radio-Frequency Electromagnetic Field Radiation (RF-EMFR) Generated by Mobile Phone Base Stations with Glycated Hemoglobin (HbA1c) and Risk of Type 2 Diabetes Mellitus. *Int. J. Environ. Res. Public Health*, 2015, *12*, 14519–14528

**DOI:** 10.3390/ijerph13030262

**Published:** 2016-02-26

**Authors:** Sultan Ayoub Meo, Yazeed Alsubaie, Zaid Almubarak, Hisham Almutawa, Yazeed AlQasem, Rana Muhammed Hasanato

**Affiliations:** 1 Department of Physiology, College of Medicine, King Saud University, P.O. Box 2925, Riyadh 11461, Saudi Arabia; yazeed-1992-@hotmail.com (Y.A.); z-almubarak@hotmail.com (Z.A.); hisham-001@hotmail.com (H.A.); yazeed.alq@gmail.com (Y.A.); 2 Department of Clinical Bio-Chemistry, College of Medicine, King Saud University, P.O. Box 2925, Riyadh 11461, Saudi Arabia; rhasanato@ksu.edu.sa

We highly appreciate the readers’ interest [1] in our article [2] titled “Association of Exposure to Radio-Frequency Electromagnetic Field Radiation (RF-EMFR) Generated by Mobile Phone Base Stations with Glycated Hemoglobin (HbA1c) and Risk of Type 2 Diabetes Mellitus” published in the International Journal of Environmental Research and Public Health [2].

In this study, we investigated the effect of exposure to radiofrequency electromagnetic field (RF-EMF) radiation emitted by mobile phone base stations (MPBSs) on the glycated hemoglobin (HbA1c) and the risk of type 2 diabetes mellitus. We found significant differences between the mean levels of HbA1c in the students who were exposed to high RF-EMFs compared to those of the students who were exposed to low RF-EMFs. Moreover, we found that the students who were exposed to high RF-EMFs had a significantly increased risk of type 2 diabetes mellitus compared to the students who were exposed to low RF-EMFs.

We are thankful to the readers for comments that “the topic of the well-structured paper authored by Meo *et al*. is challenging” [1]. We are well familiar with the various confounding factors and the variations of RF-EMF level in our daily life. In our study, we recruited the students based on their voluntary participation, apparently healthy status, similar age, same gender, nationality, regional, cultural and socio-economic status. Initially, we invited 250 participants (125 from school-1, and 125 from school-2). After detailed interviews, clinical history and examination, we carefully assessed the students whether to include them in the study or not. All the students were questioned about anthropometric parameters, age, height, weight, ethnicity, socio-economic status, and family history of diabetes mellitus, blood diseases, and cigarette smoking. After clinical history and examination, finally, we selected 159 apparently healthy male, volunteer students (96 from school-1, and 63 from School-2). Our exclusion criteria was based on generally acceptable standards and the students with known cases of gross anemia, blood diseases, history of blood transfusion, personal or family history of known diabetes mellitus, students who suffered from marked obesity, asthma, and students who smoked tobacco were excluded from the study. Moreover, students who lived close to any high transmission lines or MPBS and students who frequently consumed fast food and excess desserts and sweets were also excluded from the study. We believe that, after such well-defined exclusion criteria, there are very little chances of impact of the variations of RF-EMF level.

As per second query, we understand that, nowadays, students are frequently using mobile phones. We wish to inform that, while interviewing the potential subjects of the study, we asked the questions on the exposure to different EMF sources at their homes, living (residence) close to the any high transmission lines or MPBS. In the Kingdom of Saudi Arabia, the use of mobile phones has been strictly prohibited in the school premises. The query regarding “Wireless Fidelity (Wi-Fi) routers and their extensive use in all houses (residents of small apartments are usually exposed to radiations emitted by several Wi-Fi routers)”. In our study, we had considered the socio-economic factors and all the participants belonged to the same socioeconomic background and were mainly residing in villas rather than small apartments.

We had already mentioned few study limitations. We hope, in future research community would further conduct large sample sized studies in both genders to confirm our findings and reach at a better conclusions. Moreover, concerns raised by the reader about various confounding factors that were identified in terms of radiation exposure from (Wi-Fi) routers, smartphones and other electronic gadgets could be further addressed in future studies. Once again, we must say that, we cannot deny the services provided by the mobile phone industry but we also strongly believe that health is more important and it cannot be compromised over anything. Thus, it must be kept in mind that the mobile MPBS should not be installed in the thickly populated areas, especially in or near the school buildings. We believe, these explanations are sufficient for the better understandings of the study.
